# Unveiling the metabolic landscape of pulmonary hypertension: insights from metabolomics

**DOI:** 10.1186/s12931-024-02775-5

**Published:** 2024-05-28

**Authors:** Huixue Ba, Yingfan Guo, Yujie Jiang, Ying Li, Xuejing Dai, Yuan Liu, Xiaohui Li

**Affiliations:** 1 Department of Pharmacology, Xiangya School of Pharmaceutical Sciences, Central South University, Changsha, China; 2 Department of Pharmacy, Jiangxi Provincial People’s Hospital, The First Affiliated Hospital of Nanchang Medical College, Nanchang, China; 3 Department of Anesthesiology, The Second Xiangya Hospital of Central South University, Changsha, China; 4 Department of Health Management, The Third Xiangya Hospital of Central South University, Changsha, China; 5 Hunan Key Laboratory for Bioanalysis of Complex Matrix Samples, Changsha, China

**Keywords:** Pulmonary hypertension, Metabolomics, Amino acid metabolism, Carbohydrate metabolism, Lipid metabolism, Nucleotide metabolism, Biomarker

## Abstract

Pulmonary hypertension (PH) is regarded as cardiovascular disease with an extremely poor prognosis, primarily due to irreversible vascular remodeling. Despite decades of research progress, the absence of definitive curative therapies remains a critical challenge, leading to high mortality rates. Recent studies have shown that serious metabolic disorders generally exist in PH animal models and patients of PH, which may be the cause or results of the disease. It is imperative for future research to identify critical biomarkers of metabolic dysfunction in PH pathophysiology and to uncover metabolic targets that could enhance diagnostic and therapeutic strategies. Metabolomics offers a powerful tool for the comprehensive qualitative and quantitative analysis of metabolites within specific organisms or cells. On the basis of the findings of the metabolomics research on PH, this review summarizes the latest research progress on metabolic pathways involved in processes such as amino acid metabolism, carbohydrate metabolism, lipid metabolism, and nucleotide metabolism in the context of PH.

## Introduction

Pulmonary hypertension (PH) is a rare and devastating cardiovascular disease characterized by a markedly unfavorable prognosis. The disease course is characterized by the gradual development of pulmonary vascular remodeling, which ultimately culminates in right heart failure and mortality [1, 2]. PH is characterized by intricate alterations in signal transduction pathways. Disrupted TGF-β signaling, an enhanced endothelin axis, and attenuated NO-GC-cGMP-PDE5 signaling collectively contribute to vascular remodeling and heightened pulmonary vascular resistance. Weakened BMPR-II/SMAD signaling impedes antiproliferative signal transduction, further exacerbating PH. Elevated Notch 3 signaling promotes abnormal cell proliferation. Impaired PPARγ/ApoE signaling disrupts metabolic balance, while heightened Notch signaling fosters abnormal cell proliferation. Activation of the STAT3/NFAT pathway stimulates cellular proliferation and intensifies inflammatory responses. Simultaneously, increased hypoxia-inducible factor 1-alpha (HIF1-α) signaling in response to hypoxia induces angiogenic and proliferative changes [3]. PH is not only a pulmonary vascular disease but also a significant metabolic disorder. Recently, a growing body of research has shown that PH is considered to be a systemic disease associated with metabolic dysfunction [4, 5]. Accumulating evidence indicates multiple metabolite abnormalities in patients with PH [6]. Zhao et al. observed disrupted glycolysis, upregulated tricarboxylic acid (TCA) cycle activity, and alterations in the oxidative pathway among individuals with severe PH [7]. Lewis et al. identified indoleamine 2,3-dioxygenase as a significant plasma metabolite biomarker for PH, and established its association with right ventricular and pulmonary vascular (RV-PV) dysfunction [8].

Furthermore, patients with PH exhibit metabolic reprogramming similar to malignant tumors [9–14]. Therefore, there has been constant evolution in the corresponding research methods, leading to a more diverse exploration of diseases and the provision of more insightful findings. Traditional metabolic studies often rely on simplified methods in which the role of specific metabolic pathways in cardiovascular disease is studied narrowly. The development of discovery-based science “omics” tools (referring to genomics, transcriptomics, proteomics, and metabolomics), enables the study of molecular changes across different disease states [15]. Therefore, metabolomics studies can provide important insights into the pathogenesis of cardiovascular disease and new potential cardiovascular disease biomarkers.

In recent years, many researchers who have focused on metabolomics have found significant metabolic abnormalities in patients with PH. This review aims to summarize the progress of current research on metabolic dysfunction in PH and to provide valuable insights for subsequent research in this field.

## Amino acid metabolism

### Aminomalonic acid

Aminomalonic acid (Ama) was initially isolated from proteins in Escherichia coli and atherosclerotic plaques and identified by Buskirk [16]. Ama also mediates free radical oxidation of amino acid residues in proteins (mainly glycine and cysteine) [17]. Metabolomics studies reported that Ama is significantly increased in the plasma of patients with large aneurysms [18], diabetic patients [19] and rat models [20]. This indicates that Ama may play a role in cardiovascular diseases with inflammatory/oxidative stress components. Notably, Bujak et al. [21] found a substantial increase of Ama in the plasma of PH patients by using GC-MS. In recent years, there has been increasing evidence of inflammation and oxidative stress in the pathophysiology of PH [22]. So the rise of Ama may be closely related to inflammation and oxidative stress. Research suggests that Ama can be used as a potential biomarker of inflammation/oxidation in PH. However, the precise mechanism underlying the elevation of Ama in the plasma of PH patients remains largely unknown. Exploring this mechanism through additional experiments could offer exciting prospects for understanding the role of Ama in PH.

### Arginine

Arginine is a key intermediate in the remodeling of extracellular matrices and the production of polyamines, nitric oxide (NO), and collagen. The arginine/NOS(NO synthase)/NO pathway is important for regulating vascular tone and remodeling in PH. NO plays a significant role in reducing smooth muscle cell proliferation and promoting apoptosis or autophagy signaling, thereby limiting the progression of vascular lesions and remodeling of the vessel wall [23, 24]. Metabolomics analysis by Zhao et al. revealed that arginine levels are significantly lower in PH tissues than in normal lung tissues, but related creatine, ornithine, and urea levels are elevated [6]. These findings indicate that in the pathological state of PH, the human body uses arginine to synthesize other intermediates, such as NO, ornithine, and urea. Asymmetric dimethylarginine (ADMA) is synthesized by the methylation of arginine residues in proteins, and is an endogenous inhibitor of NOS. A lower arginine-to-ornithine ratio and higher ADMA level were shown to be associated with greater severity and mortality in PH patients with sickle cell disease [25] and systolic heart failure [26]. Furthermore, polyamines such as putrescine, spermidine, and spermine were elevated. The elevation of these proteins reflects the proliferative phenotype of various cells in the PH lung, because polyamines are needed for DNA synthesis and cell growth. Polyamines are essential for structural remodeling and sustained elevation of pulmonary artery pressure. The dominance of polyamine transport over de novo synthesis in regulating lung vascular cell polyamines during hypoxia implies that targeting polyamine transporters may be an effective intervention strategy [27]. Additionally, Zhao et al. showed that the relationship between arginine and collagen synthesis, i.e., reduced arginine levels, is associated with increased collagen production in PH lung tissue, which may promote vascular proliferation and remodeling. Additionally, the expression of genes encoding proteins such as procollagen-lysine, 2-oxoglutarate 5-dioxygenase 2, collagen type XIV alpha 1, and collagen type III alpha, which are involved in collagen synthesis, was significantly increased. Lewis et al. revealed that the level of arginine metabolites was correlated with right ventricular-pulmonary vascular dysfunction in PH [8]. Additional experiments should be conducted to fully understand the mechanisms underlying the role of arginine in PH.

### Glutamate

Glutamine is the most abundant free amino acid in plasma. It can be hydrolyzed to glutamate and converted to α-ketoglutarate (α-KG), which is involved in the TCA cycle, and it facilitates protein and lipid synthesis and providing cellular energy. In addition to the Warburg effect, metabolic disorders in PH may also lead to fatty acid oxidation and increased levels of glutaminase [9, 28, 29]. Dumas SJ et al. revealed the maladjustment of the glutamate/glutamine-N-methyl-D-aspartate receptor (NMDAR) axis in PH patients and also identified vascular NMDARs as potential targets for therapeutic intervention in PH [30]. A significant increase in circulating glutamine levels has been demonstrated in PH patients [31] as has increased glutamine metabolism in pulmonary artery endothelial cells (PAECs) [32].

Among the potential candidate pathways, the hypoxia-inducible factor 1-alpha (HIF1-α) pathway stands out as the most relevant to PH and glutamine metabolism. Dysregulated metabolism of glutamine through a mitochondrial reductive pathway is a key component of the metabolic profile observed in proliferating cells under hypoxic conditions, as well as in the context of HIF1-α-induced metabolic reprogramming [33, 34]. Activation of glutaminolytic enzymes promotes vascular remodeling in animal models of PH. On the other hand, increased glutaminolysis induces fibrosis, stimulates excessive cell proliferation, and triggers extracellular matrix migration [29]. The transglutaminase 2 (TG2) inhibitor has the potential to prevent the development of PH. However, anti-glutamine drug treatment targets have shown undesirable adverse effects [6].

Nevertheless, NMDAR show show promise, so drugs targeting these receptors deserve further exploration. Basic research combined with metabolomics research has gradually explored the mechanisms involved in increasing glutamate levels and their consequences in PH.

### Methionine

Methionine serves as a precursor for various crucial compounds, including succinyl-CoA, homocysteine, cysteine, creatine, and carnitine. Methionine undergoes conversion to S-adenosylmethionine (SAM), a highly active molecule that serves as a methyl donor. SAM can be metabolized to homocysteine (Hcy). Hcy can be remethylated to methionine. SAM can be converted to adenosine, subsequently regenerating methionine [35]. Recent research has highlighted the regulatory role of methionine in the synthesis of polyamines and glutathione. It has been reported that PH patients exhibit methionine metabolism dysregulation [31]. In a hypoxia-induced PH rat model, the methionine metabolism pathway was the top-ranked pathway. Zhao et al. reported downregulated levels of homoserine and methionine, while adenosine was upregulated. Notably, the second pathway was the betaine metabolism pathway [36]. As is well known, betaine functions as a methyl donor in the methionine cycle. Betaine has been identified as a potential biomarker, and elevated plasma betaine levels are linked to PH severity [3, 37]. However, betaine can improve PH by inhibiting SMC proliferation and exerting anti-inflammatory effects [38, 39]. Therefore, the elevation in betaine levels may not be a cause but rather a consequence of disease progression. This phenomenon could be related to the accumulation of low methylation in vivo due to betaine serving as a methyl donor for methionine or acting as an osmolyte [40].

### Aromatic amino acids

#### Tryptophan

The decarboxylation of tryptophan results in the formation of serotonin (5-HT) [41]. It has been suggested that serotonin can enhance pulmonary arterial smooth muscle cells (PASMCs) proliferation, vasoconstriction, and microthrombus formation [42]. Over 95% of free tryptophan serves as the degradation substrate for the tryptophan-kynurenine pathway, which is mediated by the rate-limiting enzymes indoleamine 2,3-dioxygenase (IDO) and tryptophan 2,3-dioxygenase (TDO) [43]. Lewis GD et al. discovered significant connections between RV-PV dysfunction and IDO [8]. The overexpression of IDO in the pulmonary endothelium effectively attenuates vascular structural remodeling in PH [10]. Moreover, tryptophan hydroxylase 1 (TPH1) expression is increased in the lung and endothelial cells of patients with idiopathic PH [44]. Additionally, the selective knockdown of PAEC-TPH1 attenuates hypoxia-induced PH in rats [45]. Bujak et al. reported decreased tryptophan levels in PH patients [21]. They believe this difference may be an indicator of metabolic conversion to serotonin, which enhances cell proliferation and vascular remodeling in PH. Coincidentally, Chen et al. also reported significantly lower tryptophan levels in individuals with PH associated with congenital heart disease (CHD-PH) than in their healthy counterparts [46]. An elevated concentration of kynurenine accompanied by immune dysfunction is associated with an unfavorable clinical prognosis in patients with PH [47]. Rhodes et al. showed elevated levels of kynurenine in PH patients compared with healthy patients, but the levels of tryptophan and serotonin did not significantly change [31]. Nagy et al. showed elevated levels of kynurenine, which is strongly associated with PH, but tryptophan was significantly decreased in idiopathic pulmonary arterial hypertension(IPAH) lungs [48]. In brief, there is great room for exploring the relationship between PH and tryptophan.

#### Phenylalanine/tyrosine

Phenylalanine is an essential amino acid that is primarily oxidized to tyrosine. It collaborates with tyrosine in the synthesis of crucial neurotransmitters and hormones such as dopamine, adrenaline, and thyroxine. Chen et al. reported increased levels of phenylalanine in patients compared with healthy controls [46]. This difference is significant because an increase in the ratio of phenylalanine/tyrosine is considered to be a biochemical marker of endothelial dysfunction [49]. Imatinib is a tyrosine kinase inhibitor that inhibits cellular proliferation and promotes apoptosis. Clinical trials have suggested that imatinib may be efficacious as an add-on therapy in PH [50].

## Carbohydrate metabolism

### Metabolic reprogramming

Glycolysis and aerobic oxidation of glucose are usually coupled, and occur in proportion to each other. The final products of glycolysis are ATP and pyruvate. Pyruvate enters the mitochondria via the mitochondrial pyruvate transporter (MTP) and acts as a substrate for pyruvate dehydrogenase (PDH), which regulates glucose oxidation and provides acetyl-CoA to the TCA cycle [51]. Carbohydrate metabolism plays an important role in the vascular remodeling of PH [52]. Moreover, the effect of impaired glucose metabolism on the rapid proliferation of PASMCs and PAECs has been confirmed in MCT-induced PH [53]. Zhao et al. used metabolomics to study lung tissue samples and reported higher levels of glucose, fructose, sorbitol, and fructose-6-phosphate in the lungs of PH patients than in those of controls. Excess glucose is utilized by the sorbitol pathway [7]. Various glycolysis intermediates, including fructose 1,6-diphosphate, 3-phosphoglycerate, and phosphoenolpyruvate, were reduced, in dicating the disruption of glycolysis in PH [7].

To better explain these changes better, researchers have used basic research to detect changes in glycolysis-related enzymes and found that the expression of the gene encoding the glucose − 6- phosphatase subunit C3(G6PC3) and the G6PC3 protein was significantly reduced in the PH lung. The expression of lactate dehydrogenase B (LDHB) and phosphofructokinase genes are increased in PH. During hypoxia, LDHB catalyzes the conversion of pyruvate to lactic acid. Bujak et al. also reported that lactate levels were significantly elevated in patients with PH, suggesting a shift in glucose metabolism toward glycolysis. This metabolic alteration offers a rapid pathway for ATP production while preventing excessive ROS production [21]. Chen et al. replicated this finding and additionally observed elevated mRNA and protein expression of pyruvate dehydrogenase kinase-1 (PDK-1) in the lungs of monocrotaline (MCT)-induced pulmonary hypertension (PH) rats. They also noted an upregulation of lactate dehydrogenase A (LDHA) at the transcriptional and translational levels in both the lungs and hearts of MCT-induced PH rats [46]. The results of Zhao et al. showed no significant change in lactate levels. The difference between the findings of Zhao et al. and Bujak et al.could be because Zhao et al. used lung samples (local metabolome) instead of plasma (systemic metabolome) from patients with PH. Among the 19 ALDH isoenzymes analyzed, aldehyde dehydrogenase 1 family member A3 (ALDH1A3) was the sole gene found to be significantly upregulated in transcriptomic analyses of PASMCs from patients with PH compared to controls. Targeted inhibition of ALDH1A3 in PASMCs holds great potential for effective therapeutic interventions in treating of PH [54]. In brief, these findings indicate that reprogramming of carbohydrate metabolism occurs in patients with severe PH, leading to impaired glucose uptake and altered glycolysis.

### The miR-124/PTBP1/PKM2 axis

Current research on PH and carbohydrate metabolism has shed light on changes in metabolites and their related enzymes. One notable finding is the miR-124/PTBP1/PKM2 pathway, which has been found to increase glycolysis in blood outgrowth ECs of patients with idiopathic PH and heritable PH [51]. MiR-124 was found to be one of the most downregulated miRNAs, leading to the upregulation of polypyrimidine-tract-binding protein 1(PTBP1), resulting in increased expression of pyruvate kinase muscle isoform 2 (PKM2), which is associated with cell proliferation and glycolysis. Moreover, restoring of normal levels of PKM2 was achieved by upregulating miR-124 or downregulating of PTBP1. Disruption of miR-124 and PTBP1 was also observed in PH Sugen-hypoxic rats [55]. Zhang et al. observed an elevated PKM2/PKM1 ratio in human pulmonary artery fibroblasts relative to that in the control cohort. Furthermore, restoration of the PKM2/PKM1 ratio in fibroblasts was accomplished via the overexpression of miR-124 or the knockdown of PTBP1, leading to the reversal of the glycolytic phenotype [56]. Another interesting finding is that peroxisome proliferator-activated receptor γ (PPARγ) suppresses TGFβ1-induced mitochondrial activation and reduces the expression of the glycolytic rate-limiting enzyme PFKP, indicating that PPARγ acts as a protective regulator in PH [57]. It was recently reported that PKR-like ER kinase (PERK) is involved in the regulation of platelet-derived growth factor receptor β (PDGFRβ) protein expression in PASMCs. Moreover, inhibiting PERK could be a promising therapeutic approach for treating PH by suppressing the PDGFRβ-signal transducer and activator of transcription 1 (STAT1) -Kruppel-like factor 4 (KLF4) -HIF1α signaling pathway and glycolysis [58]. Pulsatile shear stress (PS) downregulates glycolysis in ECs through upregulation of glucokinase regulator protein (GCKR) expression mediated by KLF4 [59]. The role of miR-124-PTBP1/PKM2-driven metabolic reprogramming in PH warrants further investigation.

## Lipid metabolism

### Sphingolipids

Sphingomyelin, the most abundant subclass of sphingolipids, plays a crucial role in transmembrane signaling as a component of cell membranes [60]. It was recently reported that multiple phosphatidylcholine and sphingomyelin lipids in plasma are significantly downregulated in PH patients [31]. Similarly, Bujak et al. reported significantly reduced plasma levels of sphingosine in patients with PH. These studies suggested that these changes in sphingolipid levels may affect fatty acid oxidation, lipid metabolism, and glucose homeostasis [21], but the underlying reasons for these changes still require further investigation.

Notably, Zhao et al. reported upregulated levels of galactosylsphingosine, sphinganine, sphingosine, and palmitoyl sphingomyelin metabolites in PH lung tissue, while sphingolipids were relatively maintained in normal lung tissue [6]. Moreover, Zhao et al. performed a gene array analysis and reported that the gene expression of N-acylsphingosine amidohydrolase, neutral sphingomyelinase activation-related factors, and sphingomyelin synthase 2 was also increased in PH patients, and was positively correlated with increased levels of sphingomyelin. Chen et al. reported that the expression of sphingosine kinase 1 (SphK1) and sphingosine-1-phosphate (S1P) is upregulated in patients with PH and in experimental PH models. In addition, inhibiting the expression of SphK1 has been shown to prevent PH. Furthermore, S1P promotes the proliferation of PASMCs by binding to S1P receptor 2 (S1PR2) and activating the ERK pathway [61]. In contrast, Rhodes and Bujak et al. reported a reduction in the level of sphingosine. This difference may have resulted from an increase in SphK1, which can convert sphingosine to S1P. Under hypoxic conditions, SphK1 activity is stimulated and can regulate the accumulation of HIF-1α through the phosphorylation of Akt/GSK3β and reactive oxygen species, as shown by Ader and colleagues [62]. PDGF stimulation induces SphK1/S1P signaling and promotes cell proliferation in PASMCs [63]. Sphingolipids are novel potential therapeutic targets for treating PH, warranting further investigation.

### Dicarboxylic fatty acids

Fatty acids can be functionalized into dicarboxylic fatty acids via terminal CH bond oxygenation and/or internal oxidative cleavage of the carbon skeleton [64]. Zhao et al. conducted metabolic analyses and observed a significant increase in the levels of tetradecanedioate, hexadecandioate, and octadecanedioate levels in PH lung tissue. These substances are all dicarboxylic fatty acids, indicating the role of the fatty acid metabolic pathway in PH [7]. Furthermore, they found that the gene encoding the aldehyde dehydrogenase 18 family member A1 (ALDH18A1), a major oxidase involved in ɷ oxidation, was overexpressed in the PH lung. Accordingly, the results of immunohistochemistry results showed that ALDH is highly expressed in SMCs and ECs of human pulmonary arteries. Briefly, both metabolomics and genetic studies have suggested that when β-oxidation is insufficient to provide ATP as a significant energy source for vascular remodeling in PH, ɷ oxidation may constitute an alternative pathway for accessing fatty acids. Perhaps ALDH can be used as a therapeutic target, bringing hope to patients with PH.

### Fatty acid amides

Fatty acid amides (FAAs) are a class of endogenous signaling lipids. They can interact with vanilloids, cannabinoids, and peroxisome proliferator-activated receptors and subsequently regulate a variety of physiological functions such as cardiac function, vasodilation, and inflammation [65]. PH patients exhibit a significant increase in circulating free fatty acids and long-chain acylcarnitines, which are associated with impaired fatty acid oxidation and subsequent accumulation of fatty acids and lipotoxicity in the myocardium [66]. Bujak et al. reported a significant downregulation of stearamide, palmitamide, and oleamide in the plasma of PH patients. The authors speculated that the downregulation of FAAs may lead to some metabolic disorders in PH, but the specific underlying mechanisms were not mentioned. Sutendra et al. reported that mice lacking the malonyl–coenzyme A(CoA) decarboxylase (MCD) do not develop hypoxia-induced PH. The absence of MCD-increasing malonyl-CoA leads to the suppression of fatty acid oxidation, which subsequently enhances glucose oxidation and prevents the metabolic shift toward glycolysis [28]. Previous studies have revealed that the activation of fatty acid synthase (FAS) in HPASMCs and HPAECs contributes to vascular remodeling and metabolic dysfunction. Furthermore, inhibiting FAS has been shown to have a protective effect [67, 68]. The cardiac lipid metabolism abnormalities associated with PH are reversed by the PPARγ agonist pioglitazone, suggesting that targeting PPARγ activation to restore fatty acid oxidation may be a therapeutic approach for treating PH [69].

### Carnitine

Carnitine is an amino acid-like compound that promotes the conversion of fat into energy. Via human plasma metabolomics, Chen et al. found that acetyl-L-carnitine, L-carnitine, and several long-chain acylcarnitines including palmitoylcarnitine, decenoylcarnitine, oleylcarnitine, linoelaidylcarnitine,trans-2-dodecenoylcarnitine and cis-5-tetradecenoylcarnitine were significantly more abundant in the in peripheral blood of patients with PH than in that of healthy control participants [46]. Increased peripheral carnitine and acylcarnitine levels may reflect the inhibition of mitochondrial fatty acid β-oxidation during PH. In addition,, their study revealed that the expression of the lipid transporter molecule CD36 was significantly upregulated in the lungs and the hearts of MCT-induced PH; the upregulation of CD36 may be a sequelae of the metabolite contributing to the accumulation of lipids [70]. Carnitine palmitoyltransferase 1 (CPT1) was shown to be highly expressed in the lungs and pulmonary arteries of rats with monocrotaline-induced PH, promoting the proliferation of PASMCs [71].

### Steroids

Dehydroepiandrosterone sulfate (DHEA-S) is a precursor of testosterone and estrogen biosynthesis. DHEA-S regulates the synthesis and secretion of vascular endothelin-1 [72, 73]. Metabolomics studies have shown that the levels of DHEA-S (dehydroepiandrosterone sulfate) and its metabolites are lower in PH patients than in healthy control subjects [31]. Studies have hypothesized that lower levels of DHEA-S may lead to the downregulation of nitric oxide and enhanced endothelin activation [74]. Nitric oxide and endothelin are two major drivers of the development of PH. Several experiments demonstrated that treatment with DHEA or DHEA-S can prevent or reverse PH in experimental rat models [75, 76]. Moreover, DHEA treatment significantly improved the 6-MWT distance and pulmonary hemodynamics in PH patients associated with chronic obstructive pulmonary disease [44].

## Nucleotide metabolism

### tRNA-specific nucleoside modifications

Transfer RNA (tRNA) is the key to efficient and accurate protein translation. However, for tRNAs to function optimally, they require extensive modifications following transcription. Further metabolome studies revealed that the levels of tRNA-specific modified nucleosides (N1-methyl inosine, N2, N2-dimethylguanosine) and circulating TCA intermediates increased in the plasma of patients with PH [31]. These modified nucleosides can be produced by the cleavage of tRNAs by angiogenin [77]. Additionally, nucleoside levels were elevated in the plasma of PH patients, and there was also an increase in angiogenin levels. Angiogenin is upregulated in cancer cells, and mediates angiogenesis, cell proliferation, and apoptosis resistance [78]. Saikia suggested that elevated levels of angiogenin and nucleotides may lead to the development of PH. Changes in tRNA quality appear to be closely related to the progression of PH.

### Adenosine monophosphate

Adenosine monophosphate (AMP) is an intermediate substance involved in the energy metabolism of adenosine triphosphate (ATP) and is an important component of the urea cycle. ATP-activated protein kinase (AMPK) is a highly conserved serine/threonine protein kinase that plays a proapoptotic role in invasive SMCs [79]. Endothelial AMPK plays protective roles against hypoxia-induced PH [80]. Zheng et al. reported that AMP levels in the PH group were significantly lower than those in the control group. In the urea cycle, ornithine and aspartate are converted into citrulline, while ATP is converted into AMP [3]. Reduced AMP levels may interrupt the citrulline-NO cycle and reduce NO expression, thereby aggravating PH. The decrease in AMP reduction indicates that AMPK may also be insufficient, which may further aggravate the disease phenotype of PH (Table 1).

**Table 1:** Detection of changes in metabolites of PH by metabolomics

Metabolic type	Name of metabolites	Sample*	Changes compared to control	Effect	References
**Amino acid**	Aminomalonic acid	Plasma	↑	Inflammation/oxidation	[21]
**metabolism**	Arginine	Lung	↓	Feedback expansion of	[6]
	Creatine	Lung	↑	blood vessels	[6]
	Ornithine	Lung	↑		[6]
	Putrescine	Lung	↑		[6]
	Spermidine	Lung	↑		[6]
	Spermine	Lung	↑		[6]
	Urea	Lung	↑		[6]
	Glutamine	Plasma	↑	Rapid cell growth	[31]
	Tryptophan	Plasma	↓	Enhancing vasoconstriction	[21, 46]
	Kynurenine	Plasma	↑		[31, 47, 48]
	Phenylalanine	Plasma	↑		[46]
	Methionine	Plasma^#^	↓		[36]
**Carbohydrates**	Glucose	Lung	↑	Impairing glucose	[7, 81]
**metabolism**	Fructose	Lung	↑	uptake and glycolysis changes	[7]
	Fructose-6-phosphate	Lung	↑		[7]
	Sorbitol	Lung	↑		[7]
	Fructose-1,6-diphosphate	Lung	↓		[7]
	3-phosphoglycerate	Lung	↓		[7]
	Phosphoenolpyruvate Lactate	Lung Plasma	↓ ↑	Increasing ATP and ROS production	[7] [21, 46]
**Lipid**	Phosphatidylcholine	Plasma	↓	Fatty acid oxidation,	[31]
**metabolism**	Sphingomyelin	Plasma	↓	lipid metabolism, and	[31]
	Sphingosine	Plasma	↓	glucose homeostasis changes	[21]
	Galactosylsphingosine	Lung	↑	PASMC proliferation	[6]
	Sphinganine	Lung	↑		[6]
	Palmitoyl sphingomyelin	Lung	↑		[6]
	Hexadecandioate	Lung	↑		[7]
	Octadecanedioate	Lung	↑	ɷ oxidation increase	[7]
	Tetradecandioate	Lung	↑		[7]
	Oleamide	Lung	↓		[21]
	Palmitamide	Plasma	↓	Metabolic disorders	[21]
	Stearamide	Plasma	↓		[21]
	L-carnitine	Plasma	↑		[46]
	Cis-5-tetradecenoylcarnitine	Plasma	↑	Blocking of	[46]
	Decenoylcarnitine	Plasma	↑	β-oxidation of fatty acid	[46]
	Linoelaidylcarnitine	Plasma	↑		[46]
	Oleylcarnitine	Plasma	↑		[46]
	Palmitoylcarnitine	Plasma	↑		[46]
	Trans-2-dodecenoylcarnitine	Plasma	↑		[46]
	DHEA-S	Plasma	↓	Down-regulate NO and enhance endothelin activation	[31]
**Nucleotide** **metabolism**	Adenosine monophosphate	Plasma^#^	↓	Reduces NO expression	[3]
	N1- Methyl inosine	Plasma	↑	Mitochondrial dysfunction	[31]
	N2, N2-dimethylguanosine	Plasma	↑		[31]

*Smaple: from human

^#^Plasma: from rat

## Research progress on other metabolic pathways

### Bile acids

Bile acids are typically synthesized in the liver and gallbladder by synthesizing 7-alpha-hydroxylase (also known as cytochrome P450 (CYP7A1)). Zhao et al. reported that the levels of bile acid metabolites such as taurochenodeoxycholate and glycochenodeoxycholate in PH lung tissue were significantly increased [82]. Moreover, they found that the expression of cytochrome P450 B1 (CYP7B1) but not that of CYP7A1, was higher in the lungs of PH patients. These results suggest that PH lung tissue may be able to synthesize bile acids [83, 84]. Corresponding basic research has shown that the CYP7B1 protein is located mainly on pulmonary vascular ECs, which further indicates that there is a complex new relationship to be studied between bile acid and PH. Conversely, it has been found that obeticholic acid (OCA) is the most advanced bile acid-derived agonist used in the clinic; this agent prevents lung diseases such as PH and pulmonary fibrosis by reducing the progression of inflammation and vascular remodeling [85]. The reasons behind these conflicting results remain to be investigated.

### Urea cycle

The urea cycle, also known as the ornithine cycle, is a biochemical process in which ammonia is converted into urea for excretion. Zheng identified that there are significant differences in the metabolites of AMP, 4-hydroxyproline, ornithine, urea, and N-acetylornithine between the PH group and the control group [3]. In the PH model, AMP was decreased, and 4-hydroxyproline, ornithine, urea, and N-acetylornithine were elevated. These results indicate that the urea cycle was disrupted. Moreover, these changes lead to the proliferation of SMCs and ECs, as well as increased collagen synthesis.

### TCA cycle

The TCA cycle is a key metabolic pathway that unifies the metabolism of carbohydrates, fats, and proteins. In general, when the cell energy supply is in an insufficient state (ADP concentration is high, ATP, NADH concentration is low), the rate of TCA cycle progression accelerates. Zhao et al. reported that most of the TCA cycle intermediates including citrate, cis-aconitate, succinate, and succinyl carnitine were significantly increased in PH lung tissue from patients with PH [7]. Furthermore, they found increased levels of related enzymes, such as iron 2 response element binding protein 1 (IREB1) and IREB2. These enzymes are different isoforms of aconitase, that catalyze the conversion of citrate to aconitate. This finding suggested increased aconitase activity in the lungs of PH patients. Furthermore, proteomic analysis of PAECs revealed reduced expression levels of solute carrier family 25A1 (SLC25A1), a mitochondrial citrate transporter that facilitates the efflux of citrate from the mitochondria to the cytoplasm [86]. Through the inhibition of PDH, HIF-1α activation effectively impedes the conversion of pyruvate to acetyl-CoA, thereby obstructing the entry of pyruvate into the TCA cycle under hypoxic conditions [87]. These findings indicate that the dysregulated expression of metabolites and related genes in the TCA cycle in PH patients may reflect mitochondrial dysfunction in PH lung tissue.

### Heme metabolism pathway

Heme metabolism consists of two distinct processes: heme synthesis and heme degradation. Heme oxidase is responsible for the conversion of heme into bilirubin. The serum bilirubin concentration is significantly elevated in PH patients and could serve as a predictor of PH mortality [88–90]. Zhao et al. [6] reported a significant difference in heme metabolism in PH samples compared with that in control group samples. Although there was no significant change in the synthesis of heme, the levels of biliverdin and bilirubin were significantly increased in the PH treatment group. An increase in bilirubin indicates an increase in hemoglobin degradation, which may indicate an increase in hemolysis in PH patients. They also performed genetic analysis of related metabolic enzymes and found that heme synthesis could be increased by increasing the expression of delta-aminolevulinate synthase 2 (ALAS2) and decreasing the expression of beta-1,3-glucuronyltransferase 3 glucuronosyltransferase I (B3GAT3). The reduction in B3GAT3 may be due to a negative feedback mechanism of bilirubin accumulation in heme metabolism. These results suggest the likely role of heme metabolism in PH.

### CtBP1

**Fig. 1:**
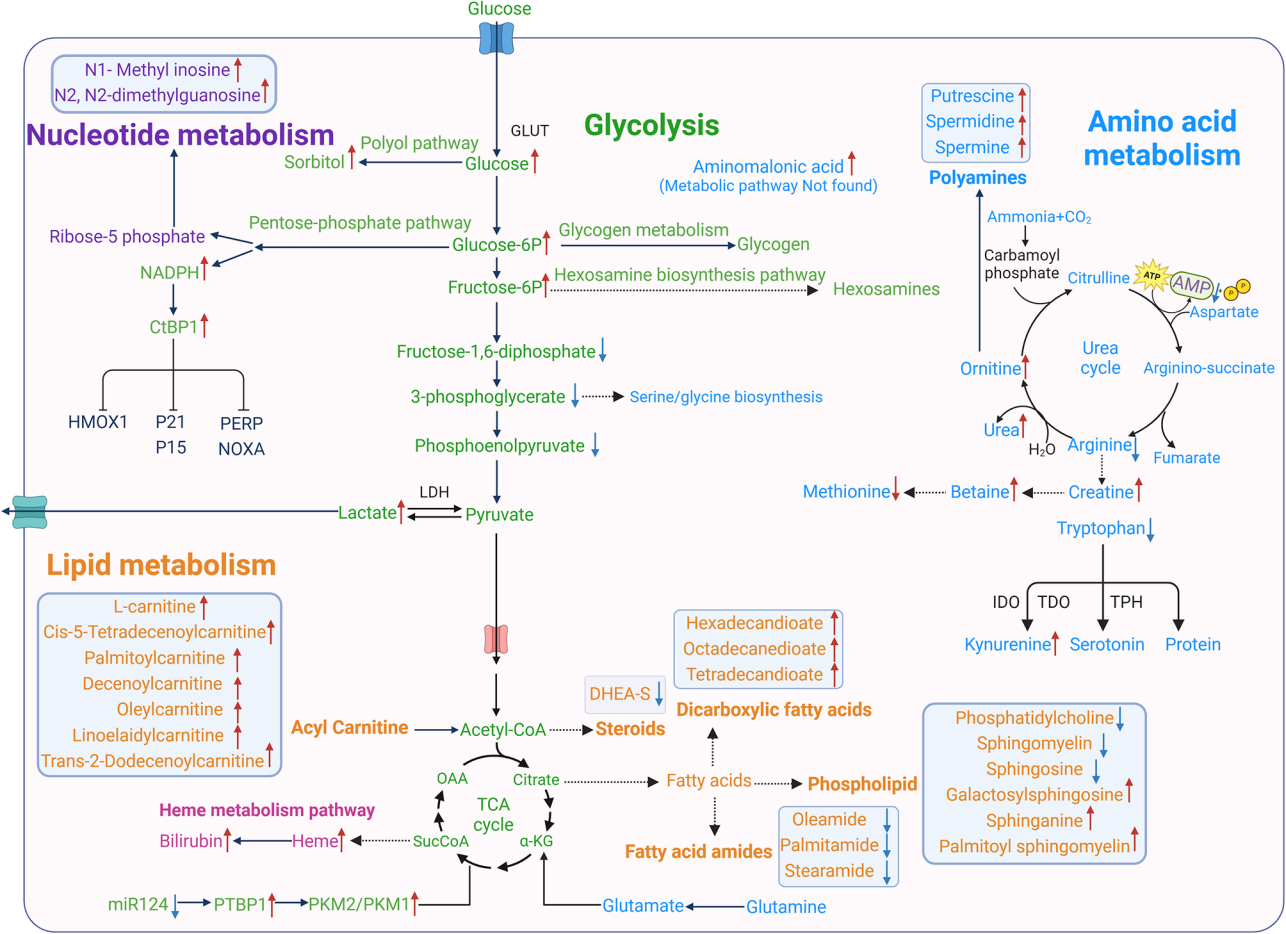
Summary of other metabolic pathway changes in pulmonary hypertension

The C-terminal binding protein (CtBP) is a dimeric transcriptional repressor encoded by two paralogous genes (CTBP1 and CTBP2), and is considered to be a cellular metabolic “sensor”. CtBP is a sensor of the reduced form of NADH [91]. CTBP is overexpressed in many cancers, including prostate cancer, ovarian cancer, colon cancer, and breast cancer [92–94]. Studies have revealed that CtBPs promote cellular survival primarily through the repression of Bcl-2 family members and other proapoptotic molecules (PERP, Bax, Bik, Puma, p21, and Noxa), as well as tumor suppressors [91, 94, 95]. Li et al. used cell metabolomics to study human PH fibroblasts and reported that the levels of free NADH and aerobic glycolysis metabolites were significantly greater in PH-Fibs than in control (CO-Fibs). They also found increased CtBP1 expression in the outer membrane fibroblasts in PH [14]. Therefore, aerobic glycolysis likely promoted an increase in the concentration of free NADH, which in turn increased CtBP1 activity. They also found that treatment with 4-methylthio-2-oxobutyric acid (MTOB), a pharmacological inhibitor of CtBP1, normalizes fibroblast proliferation, inflammation, and abnormal metabolic signaling in hypoxic mice. They employed siRNA to genetically inhibit CtBP1, which resulted in the upregulation of the cyclin-dependent genes (p15 and p21) and proapoptotic regulators (NOXA and PERP). These findings extend their previous observations of abnormal mitochondrial metabolism in PH and highlight the critical role of metabolic adaptation in supporting fibroblast proliferation and inflammatory activation [10]. Future studies should investigate whether the inhibition of CtBP1 can be combined with other vasodilatory drugs to achieve a reversal of pulmonary hypertension. Figure 1 provides an overview of the metabolic pathway changes that occur in PH patients discussed in this review.

Individual pathways and names are highlighted by specific colors, and enzymes are denoted by black fonts Dashed arrows represent multiple steps of metabolism. TCA: tricarboxylic acid cycle, PKM2/1: pyruvate kinase muscle isoforms 2/1, PTBP1: polypyrimidine tract binding protein 1, CtBP1: C-terminal binding protein 1, AMP: adenosine monophosphate, GLUT: glucose transporter, α-KG: α-ketoglutarate, LDH: lactate dehydrogenase, MCT: monocarboxylate transporter, NADPH: nicotinamide adenine dinucleotide phosphate, IDO: indoleamine 2,3-dioxygenase, TDO: tryptophan 2,3-dioxygenase, TPH: tryptophan hydroxylase.

## Discussion

PH is a group of diseases still characterized by high morbidity and mortality, and the urgent clinical needs associated with these diseases have still not been properly addressed. In this review, we propose a comprehensive metabolomic analysis to investigate the disturbed metabolic features of PH. Metabolic rewiring in PH is characterized by increased glycolysis, reduced glucose oxidation phosphorylation, mitochondrial dysfunction, disrupted fatty acid oxidation, and abnormal amino acid metabolism. These alterations collectively contribute to energy metabolism dysregulation, providing new insights into the pathogenesis of PH. Relevant metabolic studies can explore PH-related diseases not only through the changes in the pulmonary artery but also through the expansion of metabolic studies of the whole body, which may lead to great prospects for the treatment of diseases and the development of new drugs.

Metabolomics, a comprehensive analysis of small molecule metabolites, has been increasingly recognized as a technique to for understanding the pathophysiology and differentiating different stages of PH, and has emerged as a powerful tool for investigating the metabolic alterations associated with PH [8, 31]. Technological progress is driving the development of more sensitive and high-throughput analytical techniques for metabolomic profiling. Notably, advances in mass spectrometry (MS) and nuclear magnetic resonance spectroscopy (NMR) have significantly improved the detection and quantification of metabolites in metabolomics research [96]. High-throughput MS imaging (MSI) has emerged as a powerful tool for visualizing the spatial distribution of small metabolite molecules. Progress in data analysis methods is crucial for extracting meaningful information from the vast amount of metabolomic data. Machine learning techniques, including pattern recognition, clustering, and classification algorithms, are employed to identify disease-specific metabolic signatures and predict disease outcomes.

Glycolysis occupies a central position in the overall metabolic network and is connected to other metabolic pathways through substrates, products, and intermediates. The pentose phosphate pathway plays a pivotal role in cellular metabolism by acting as a crucial bridge between glycolysis and other metabolic pathways, as it provides nicotinamide adenine dinucleotide phosphate (NADPH) and ribose-5-phosphate, which are involved in synthesizing nucleotides and lipids. PH patients have been shown to exhibit impaired TCA cycle activity and dysregulated mitochondrial function [8]. Moreover, integrating metabolomics with other omics technologies, such as genomics, proteomics, and transcriptomics, can provide a more holistic understanding of disease mechanisms. Epigenetic changes, such as DNA methylation and histone modifications, can act as upstream regulators of altered metabolism by influencing the expression of genes involved in metabolic pathways. Studies have shown that the expression of HIF1-α in the pulmonary endothelium is increased in hypoxia-induced PH [97], and less severe PH and vascular remodeling occur in HIF knockout mice under chronic hypoxia [98–100], suggesting that HIF-1α is involved in hypoxia-induced vascular remodeling and PH formation. ELAMAA reported that the PHD/HIF-1α pathway can participate in the development of PH [101], and PHD2 deletion in endothelial cells and arterial smooth muscle cells increases pulmonary systolic blood pressure, which in turn increases right ventricular pressure. Under hypoxic conditions, the expression of miRNA-17/miRNA-20a is upregulated, and targeted inhibition of PHD2 can increase the stability of HIF-1α, thus promoting pulmonary vascular remodeling [102]. In addition, PHD2/HIF-1α also acts on the glycolytic enzyme 6-phosphofructose-2-kinase/fructose-2,6-bisphosphatase 3 in endothelial cells, thereby enhancing the adaptability of the right ventricle in a hypoxic state [103]. HIFs play a vital role in regulating metabolic enzymes like PDK and PDH, thereby influencing metabolic shifts and mitochondrial dynamics. By integrating metabolomic data with other omics disciplines, such as genomics and proteomics, we can gain a more comprehensive understanding of the molecular pathways involved in the development of PH [104].

Mutations in the BMPR2 gene can result in defects in BMP signaling pathway function, which in turn affects the normal development and function of pulmonary blood vessels. BMPR2 mutations not only affect glycolytic reprogramming but also involve abnormalities in the TCA cycle, polyamine, and sphingolipid metabolism pathways. BMPR2 gene mutations may regulate metabolic reprogramming through abnormal activation of the TGF-β/SMAD signaling pathway. Increased TGF-β/SMAD signaling transduction may lead to increased β-catenin/Wnt signaling and downregulation of PPAR-γ, which may promote glycolysis [105]. Recent studies suggest that targeting metabolic pathways could be a new direction for treating PH. Therapies focused on glucose metabolism, such as dichloroacetate, an inhibitor of pyruvate dehydrogenase kinase, have been found to decrease pulmonary arterial pressure and vascular resistance. Additionally, modulating fatty acid metabolism may also have therapeutic benefits for PH, as drugs such as trimetazidine and ranolazine reduce long-chain fatty acid oxidation to improve cardiac output. These findings highlight the potential of targeting metabolic pathways as a novel strategy for treating PH [96].

In addition, most related studies involve smaller cohorts [8, 21, 31], which may limit the accuracy of diagnosing these diseases and providing a comprehensive understanding of metabolic alterations. To address this limitation, it is imperative to establish a sample repository for PH that encompasses a larger cohort dataset obtained from multicenter studies. A large sample repository has the advantages of providing better stability, lower variability, greater regularity, and greater reliability. Furthermore, establishing a specialized sample repository for PH would also promote collaborative research and data sharing. By integrating data from multiple research centers, a more comprehensive analysis of metabolic pathway alterations can be conducted, leading to the discovery of new biomarkers and potential therapeutic targets associated with PH.

## Data Availability

No datasets were generated or analysed during the current study.
